# Safety analyses from the phase 3 ASCENT trial of sacituzumab govitecan in metastatic triple-negative breast cancer

**DOI:** 10.1038/s41523-022-00467-1

**Published:** 2022-08-29

**Authors:** Hope S. Rugo, Sara M. Tolaney, Delphine Loirat, Kevin Punie, Aditya Bardia, Sara A. Hurvitz, Joyce O’Shaughnessy, Javier Cortés, Véronique Diéras, Lisa A. Carey, Luca Gianni, Martine J. Piccart, Sibylle Loibl, David M. Goldenberg, Quan Hong, Martin Olivo, Loretta M. Itri, Kevin Kalinsky

**Affiliations:** 1 Department of Medicine, University of California San Francisco Helen Diller Family Comprehensive Cancer Center, San Francisco, CA USA; 2 Department of Medical Oncology, Dana-Farber Cancer Institute, Boston, MA USA; 3 Department of Medical Oncology and D3i, Institut Curie, Paris, France; 4 Department of General Medical Oncology, University Hospitals Leuven, Leuven, Belgium; 5 Department of Hematology/Oncology, Massachusetts General Hospital Cancer Center, Harvard Medical School, Boston, MA USA; 6 Medical Oncology, University of California, Los Angeles, Jonsson Comprehensive Cancer Center, Los Angeles, CA USA; 7 Baylor University Medical Center, Texas Oncology, US Oncology, Dallas, TX USA; 8 International Breast Cancer Center (IBCC), Quiron Group, Madrid & Barcelona, Barcelona, Spain; 9 Vall d´Hebron Institute of Oncology (VHIO), Barcelona, Spain; 10 Universidad Europea de Madrid, Faculty of Biomedical and Health Sciences, Department of Medicine, Madrid, Spain; 11 Department of Medical Oncology, Centre Eugène Marquis, Rennes, France; 12 Department of Hematology and Oncology, University of North Carolina Lineberger Comprehensive Cancer Center, Chapel Hill, NC USA; 13 Gianni Bonadonna Foundation, Milano, Italy; 14 Medical Oncology, Institute Jules Bordet, Brussels, Belgium; 15 Department of Medicine and Research, Hämatologisch-Onkologische Gemeinschaftspraxis am Bethanien-Krankenhaus, Frankfurt, Germany; 16 Department of Clinical Development, Immunomedics, Inc., Morris Plains, NJ USA; 17 Columbia University Irving Medical Center, New York, NY USA; 18 Present Address: Winship Cancer Institute, Emory University, Atlanta, GA USA

**Keywords:** Breast cancer, Drug safety

## Abstract

Sacituzumab govitecan (SG) is an anti-Trop-2 antibody-drug conjugate with an SN-38 payload. In the ASCENT study, patients with metastatic triple-negative breast cancer (mTNBC) relapsed/refractory to ≥2 prior chemotherapy regimens (≥1 in the metastatic setting), received SG or single-agent treatment of physician’s choice (eribulin, vinorelbine, capecitabine, or gemcitabine). This ASCENT safety analysis includes the impact of age and *UGT1A1* polymorphisms, which hinder SN-38 detoxification. SG demonstrated a manageable safety profile in patients with mTNBC, including those ≥65 years; neutropenia/diarrhea are key adverse events (AE). Patients with *UGT1A1* *28/*28 genotype versus those with 1/*28 and *1/*1 genotypes had higher rates of grade ≥3 SG-related neutropenia (59% vs 47% and 53%), febrile neutropenia (18% vs 5% and 3%), anemia (15% vs 6% and 4%), and diarrhea (15% vs 9% and 10%), respectively. Individuals with *UGT1A1* *28/*28 genotype should be monitored closely; active monitoring and routine AE management allow optimal therapeutic exposure of SG.

## Introduction

Triple-negative breast cancer (TNBC) comprises approximately 15% of all breast cancers and is associated with a poor prognosis^1–3^. Due to absent hormone and HER2 receptors, targeted strategies used in other types of breast cancer are not effective for metastatic TNBC (mTNBC), and current treatment guidelines recommend single-agent chemotherapy for patients without known biomarkers associated with targeted therapy^2,4^. Patients with TNBC have poorer outcomes compared with those with other breast cancer subtypes, including higher and earlier risk of relapse and shorter survival^5,6^. Among patients with previously treated mTNBC receiving single-agent chemotherapy, median progression-free survival (PFS) is only approximately 2–3 months, with median overall survival (OS) of approximately 8–15 months^7–10^. Common adverse events (AEs) with single-agent chemotherapy include hematologic AEs such as neutropenia, as well as diarrhea, nausea, and alopecia^8,11^. Although TNBC is more common in younger compared with older breast cancer patients, a significant number of TNBCs arise in older patients due to an aging population^12,13^. Approximately 20% of patients diagnosed with TNBC are ≥65 years old^14,15^. Furthermore, older patients are generally less fit for chemotherapy due to a greater rate of comorbidities, increased use of concomitant medications, and the potential for greater impact on health-related quality of life^16,17^. Due to the limited survival outcomes with single-agent chemotherapy regimens and the potential for increased AEs in older patients, there is an unmet medical need for novel targeted agents for all patients with pretreated mTNBC.

Trophoblast cell surface antigen-2 (Trop-2) is a calcium signal transducer that has been linked to poor outcomes in multiple cancer types^18^. Overexpression of membrane-localized Trop-2 has been associated with poor prognosis and increased tumor growth in breast cancer^19,20^. Trop-2 is the target of sacituzumab govitecan (SG), an antibody-drug conjugate (ADC) in which the payload of SN-38, a topoisomerase-1 inhibitor and the active metabolite of irinotecan, is conjugated with an anti-Trop-2 humanized monoclonal antibody via a proprietary hydrolyzable linker^21^. This allows for cytotoxic SN-38 liberation in the acidic tumor microenvironment without prerequisite internalization and subsequent enzymatic cleavage^22^, enabling “bystander effect” tumor cell killing^23^. Upon binding, its drug-antibody ratio of 7.6:1 allows the release of a high localized SN-38 concentration from SG without adversely impacting binding or pharmacokinetics^21,24^.

The safety and efficacy of SG was assessed in a single-arm, phase 1/2 trial in 108 patients with previously treated mTNBC, and reported an objective response rate (ORR) of 33.3% (95% confidence interval [CI], 24.6–43.1) and a median progression-free survival (PFS) of 5.5 months (95% CI, 4.1–6.3), with low rates of discontinuation^23^. This trial also showed that the 10 mg/kg dose had a manageable safety profile with better efficacy than lower doses^24^. The pivotal, international, multicenter phase 3 ASCENT (NCT02574455) trial randomized 529 patients with relapsed or refractory mTNBC to receive SG or single-agent treatment of physician’s choice (TPC; eribulin, vinorelbine, gemcitabine, or capecitabine); the clinical benefit seen was consistent with the phase 1/2 study results^25^. There was a significant ORR (35% vs 5%), PFS (median 5.6 vs 1.7 months; hazard ratio [HR], 0.41; *p* < 0.001) and overall survival (OS) (median 12.1 vs 6.7 months; HR 0.48; *p* < 0.001) improvement for SG relative to single-agent chemotherapy. Key SG treatment-related adverse events (TRAEs) of grade ≥3 included neutropenia (51% vs 33%), diarrhea (10% vs <1%), anemia (8% vs 5%), and febrile neutropenia (6% vs 2%). SG was subsequently granted full approval by the US Food and Drug Administration (FDA) for patients with unresectable, locally advanced or metastatic TNBC who have received ≥2 prior systemic therapies, at least one of them for metastatic disease^26^.

Neutropenia and diarrhea are toxicities associated with irinotecan, attributable to its active metabolite, SN-38, which is the cytotoxic payload of SG^21,27^. The enzyme uridine diphosphate glucuronosyltransferase 1A1 (UGT1A1) plays a key role in detoxifying SN-38 by glucuronidation^24^, producing a metabolite that is then eliminated from the body primarily by biliary excretion^28^. UGT1A1 activity is reduced in the UGT1A1 *28/*28 phenotype, which is found in approximately 10% of North American patients; this polymorphism leads to a higher risk of neutropenia and diarrhea with irinotecan therapy^27–29^. For this reason, FDA guidelines call for a reduction in the irinotecan starting dose in patients with colorectal cancer with known UGT1A1 *28/*28 status^27,28,30^, and European Society for Medical Oncology guidelines recommend *UGT1A1* genotyping in patients with metastatic colorectal cancer for whom an irinotecan dose >180 mg/m^2^ is planned and there is a suspicion of UGT1A1 deficiency, as reflected by low conjugated bilirubin^31^.

As part of the continuing safety evaluation of SG, we conducted a detailed and updated safety analysis of AEs of interest, an analysis of time to onset and duration of key TRAEs, and examined TRAEs in older patients and by *UGT1A1* variant status.

## Results

### Patient disposition and baseline characteristics

Of 529 patients randomized in ASCENT, 9 (3%) and 38 (15%) in the SG and TPC arms, respectively, did not receive treatment (Supplementary Fig. 1). The safety population consisted of 482 patients (of whom 53 had brain metastases) who received at least one dose of study treatment, including 258 in the SG arm and 224 in the TPC arm. Among patients in the safety population who were treated with TPC, 123 (55%), 28 (13%), 32 (14%), and 41 (18%) received eribulin, capecitabine, gemcitabine, and vinorelbine, respectively. At data cutoff (March 11, 2020), 17 and 0 patients remained on treatment in the SG and TPC arms, respectively. In both arms, the primary reason for discontinuation (SG vs TPC) was disease progression (86% vs 82%).

Patient baseline characteristics were well-balanced between arms (Table 1). The median age was 54 (range 27–82), the median number of prior anticancer regimens (any setting) was 4 (range 2–17), and all patients had previously received a taxane. There were 49 (19%) and 48 (21%) patients aged ≥65 years in the SG and TPC arms, respectively; of these, 8 and 13 patients were aged ≥75 years.

**Table 1:** Patient demographics and disease characteristics at baseline (Safety Population).

Characteristic	SG (*n* = 258)	TPC (*n* = 224)
Female, *n (%)*	256 (99)	224 (100)
Age		
Median, years (range)	54 (27–82)	54 (30–81)
<50 years, *n (%)*	92 (36)	71 (32)
50–64 years, *n (%)*	117 (45)	105 (47)
≥65 years, *n (%)*	49 (19)	48 (21)
ECOG PS, *n (%)*		
0	117 (45)	93 (42)
1	141 (55)	131 (58)
Race or ethnic group, *n (%)*		
White	211 (82)	172 (77)
Black	25 (10)	31 (14)
Asian	11 (4)	9 (4)
Other	11 (4)	12 (5)
Brain metastasis at randomization, *n (%)*		
Yes	30 (12)	23 (10)
No	228 (88)	201 (90)
Median no. prior anticancer regimens^1^, (range)	4 (2–17)	4 (2–14)
Prior chemotherapy regimens from randomization stratification, *n (%)*		
2–3	178 (69)	158 (71)
>3	80 (31)	66 (29)
*BRCA1/2* mutation status^2^, *n (%)*		
Negative	145 (56)	123 (55)
Positive	19 (7)	20 (9)
Unknown	94 (36)	81 (36)
*UGT1A1* variant status^3^, *n (%)*		
*1/*1 (wild type)	113 (44)	NA
*1/*28 (heterozygous)	96 (37)	NA
*28/*28 (homozygous)	34 (13)	NA
Unknown/other	15 (6)	NA
Original diagnosis of TNBC^4^, *n (%)*		
Yes	184 (71)	156 (70)
No	74 (29)	68 (30)
Median time from metastatic diagnosis, mo. (range)	17.1 (0.1–202.9)	15.5 (−0.4–95.8)

Assessed in the safety population.

*BRCA* breast cancer gene, *ECOG PS* Eastern Cooperative Oncology Group performance status, *NA* not applicable, *SG* sacituzumab govitecan, *TNBC* triple-negative breast cancer, *TPC* treatment of physician’s choice, *UGT* uridine diphosphate glucuronosyltransferase.

^1^Anticancer regimens refer to any prior metastatic/neoadjuvant/adjuvant/locally advanced regimens used to treat an eligible breast cancer patient, including hormonal treatment.

^2^Approximately 64% of patients in each arm consented and had known *BRCA1/2* mutation status.

^3^Population of patients with known *UGT1A1* variant status was 250.

^4^Patients on study either had TNBC at initial diagnosis or had hormone receptor-positive disease that converted to hormone-negative at time of study entry.

### Safety by treatment arm

In the SG arm, median relative dose intensity was 99.7%. As reported previously, the most common TRAEs (all grades) in the SG arm were neutropenia (63%), diarrhea (59%), and nausea (57%); the most common grade ≥3 TRAEs were neutropenia (51%), leukopenia (10%), and diarrhea (10%; Supplementary Table 1)^25^. In the TPC arm overall, the most common TRAEs (all grades) were neutropenia (43%), fatigue (30%), and nausea (26%); most common grade ≥3 TRAEs were neutropenia (33%), leukopenia (5%), anemia (5%), and fatigue (5%). No patient vs 2 patients (1%) in the SG vs TPC arms had treatment-related grade ≥3 neuropathy. No patients experienced treatment-related interstitial lung disease of any grade with TPC. In the SG arm, there was 1 event (0.4%) of grade 3 pneumonitis complicated by prior radiation and progressive disease. This patient was a 52-year-old woman who experienced the event 14 days after her last dose of SG; it resolved 7 weeks after onset without sequalae^25^. SG-related grade ≥3 rash (<1%), all-grade ocular disorders (<5%), and hyperglycemia (<2%) were also infrequent. Rates of discontinuation due to TRAEs were low in both the SG (2%) and TPC (3%) arms. No treatment-related deaths were reported in the SG arm, and 1 death (neutropenic sepsis related to eribulin) was reported in the TPC arm. There were no discontinuations due to SG-related neutropenia, febrile neutropenia, or diarrhea.

An assessment of TRAEs by TPC agent (either eribulin alone or vinorelbine, capecitabine, and gemcitabine combined) showed similar key all-grade and grade ≥3 TRAEs as the TPC arm overall (Supplementary Table 1). Minor differences between the eribulin subgroup and the vinorelbine, capecitabine, and gemcitabine combined subgroup included slightly lower rates of all-grade neutropenia (39% vs 48%) and diarrhea (8% vs 17%) and grade ≥3 anemia (2% vs 8%), but higher rates of all-grade alopecia (25% vs 4%), respectively.

#### Neutropenia

**Fig. 1:**
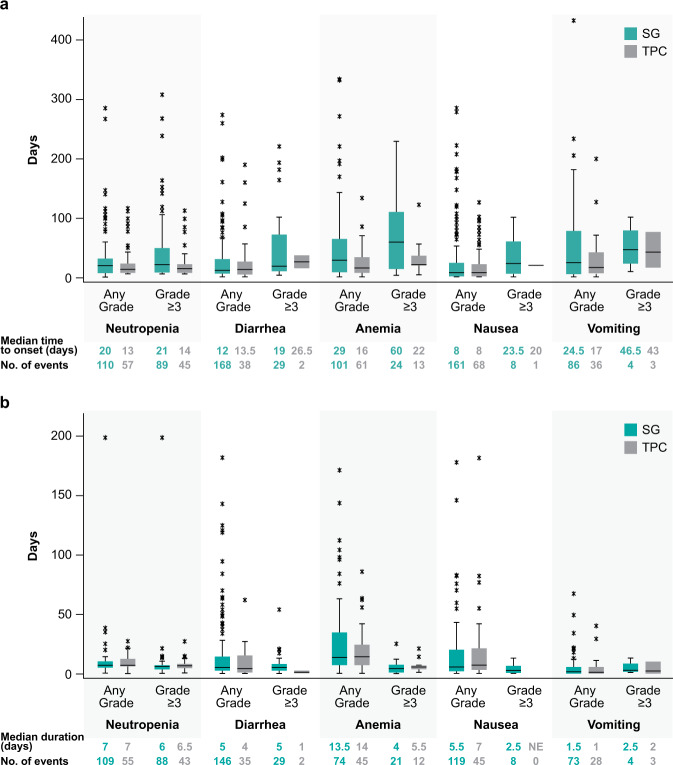
Time course of treatment-related adverse events of special interest. **a** Time to onset of first event of treatment-related AESI and (**b**) duration of an individual episode of treatment-related AESI of any grade and grade ≥3 in the Safety Population. Box and whisker plots, with upper and lower boundaries of each box plot representing the 25th and 75th percentiles and the horizontal lines within the box representing median values. Whiskers extend to the last observation if it was not an outlier (defined as greater than Q3 + 1.5 × IQR or less than Q1–1.5 × IQR) or to the minimum/maximum values if an outlier was not identified. Outliers are indicated by an asterisk. AESI adverse event of special interest, IQR interquartile range, Q1 first quartile, Q3 third quartile, SG sacituzumab govitecan, TPC treatment of physician’s choice.

**Fig. 2:**
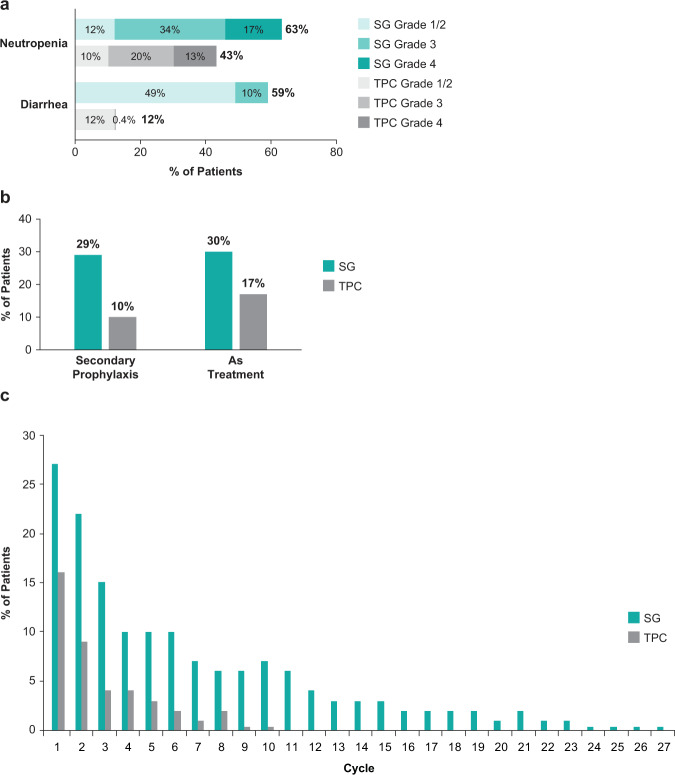
Incidence of neutropenia and diarrhea, and G-CSF use. **a** Incidence of neutropenia and diarrhea by grade, **b** use of G-CSF in the safety population, and **c** use of G-CSF by cycle in the safety population. Percent of patients using G-CSF by treatment cycle is based on the total number of patients treated in cycle 1 (SG, *n* = 258; TPC, *n* = 224). G-CSF granulocyte colony-stimulating factor, SG sacituzumab govitecan, TPC treatment of physician’s choice.

Median time to onset of the first event of treatment-related grade ≥3 neutropenia was 21 days and 14 days for the SG and TPC arms, and median duration of an individual episode of grade ≥3 neutropenia was 6.0 and 6.5 days, respectively (Fig. 1). Treatment-related neutropenia of all grades was more frequent in the SG arm compared with the TPC arm (any grade: 63% vs 43%; grade ≥3: 51% vs 33%) (Fig. 2A). In the first treatment cycle with SG vs TPC, 43% vs 33% and 28% vs 23% of patients had any grade and grade ≥3 neutropenia, respectively; 4% vs 0.4% of patients in the SG vs TPC arms had febrile neutropenia, all grade ≥3. Myeloid growth factor use was more common in the SG vs TPC arm (total use, 49% vs 23%; for secondary prophylaxis, 29% vs 10%; as treatment, 30% vs 17%; Fig. 2B), was most common in the first 3 cycles of treatment, and treatment was less common in later cycles (<10% of SG-treated patients; Fig. 2C). Dose interruptions due to treatment-related neutropenia or febrile neutropenia occurred in 46% and 21% of patients in the SG and TPC arms, respectively, whereas dose reductions for this reason occurred in 11% and 19% of patients, respectively. Treatment-related neutropenia and febrile neutropenia led to discontinuations in 0% vs 1% and 0% vs <1% of patients in the SG vs TPC arms, respectively.

#### Diarrhea

Median times to onset of the first event of treatment-related grade ≥3 diarrhea were 19 days and 26.5 days for the SG and TPC arms, and median durations of an individual episode of grade ≥3 diarrhea were 5 days and 1 day, respectively (Fig. 1). Treatment-related diarrhea of all grades was more frequent in the SG arm (any grade: 59% vs 12%; grade ≥3: 10% vs 0.4%) (Fig. 2A). Grades 1 and 2 diarrheas occurred in 30% vs 7% and 19% vs 5% of SG- vs TPC-treated patients. There were no events of grade 4 or 5 diarrhea. Concomitant medicine was used for diarrhea management in 55% vs 10% of SG- vs TPC-treated patients. In the SG vs TPC arms, 55% vs 8% received loperamide and 10% vs 2% received atropine, respectively. Dose interruptions due to treatment-related diarrhea occurred in 5% of patients in the SG arm and no patients in the TPC arm, whereas dose reductions for diarrhea occurred in 5% and <1% of patients, respectively. No treatment discontinuations due to treatment-related diarrhea occurred in either arm.

#### Nausea and vomiting

In the SG vs TPC arms, median time to onset of the first event of any grade nausea and vomiting was 8 vs 8 days and 24.5 vs 17 days after treatment initiation, respectively (Fig. 1); however, the frequencies of grade ≥3 nausea and vomiting were low (<3%). Premedication or concomitant medication for nausea and vomiting, such as 5-HT3 antagonists (e.g., ondansetron) and receptor antagonists (e.g., prochlorperazine), was used in 86% and 63% of patients in the SG and TPC arms, respectively.

#### Other adverse events

Among other TRAEs, alopecia (46% vs 16%) was more common in the SG arm. Any grade (45% vs 30%) but not grade ≥3 fatigue (3% vs 5%) was also more common in the SG arm.

### Safety outcomes in older patients

Patients aged ≥65 years represent approximately 20% of the ASCENT study population. Patients aged ≥65 or ≥75 years treated with SG generally had safety profiles similar to those aged <65 years, with a similar rate of treatment-emergent AEs (TEAEs; all-grade and grade ≥3) and TEAEs leading to dose reduction or treatment discontinuation, as well as a low rate of discontinuations due to AEs (Supplementary Table 2). Although patients aged ≥65 years who received SG had a slightly higher rate of all-grade and grade ≥3 TRAEs compared with those who received TPC (98% vs 83% and 63% vs 54%, respectively), TRAEs leading to dose reduction (35% vs 33%) were generally similar across treatment arms and there were no treatment-related deaths. Patients aged ≥65 years in the SG arm experienced higher rates of TRAEs leading to dose reduction compared with those aged <65 years (35% vs 19%), but patients aged ≥65 years in the TPC arm also had higher rates of TRAEs leading to dose reduction vs those aged <65 years (33% vs 23%). Key TRAEs leading to dose reduction in SG- vs TPC-treated older patients were neutropenia (10% vs 25%), fatigue/asthenia (10% vs 4%), diarrhea (6% vs 0%), febrile neutropenia (6% vs 0%), and nausea (4% vs 0%), respectively; patients aged <65 years had similar key TRAES leading to dose reductions, including neutropenia (9% vs 18%), diarrhea (4% vs 1%), fatigue/asthenia (2% vs 3%), and febrile neutropenia (2% vs 0%), respectively.

In patients aged ≥65 years, key grade ≥3 TRAEs (SG vs TPC) were neutropenia (45% vs 40%), anemia (14% vs 6%), leukopenia (10% vs 4%), diarrhea (10% vs 0%), and febrile neutropenia (8% vs 0%; Table 2), similar to the safety profile in the overall study population. SG-related all-grade ocular disorders and hyperglycemia occurred in 8% and 4% of older patients, respectively. No interstitial lung disease, grade ≥3 cardiovascular toxicity, grade ≥2 peripheral neuropathy, or grade ≥3 rash was reported with SG in older patients.

**Table 2:** Treatment-related adverse events of all grades reported in >20% and of grade 3 or 4 reported in >5% of patients by age.

TRAE^1^, *n (%)*	SG						TPC					
	<65 years (*n* = 209)			≥65 years (*n* = 49)			<65 years *(n* = 176)			≥65 years (*n* = 48)		
	All grades	Grade 3	Grade 4	All grades	Grade 3	Grade 4	All grades	Grade 3	Grade 4	All grades	Grade 3	Grade 4
Hematologic												
Neutropenia^2^	134 (64)	76 (36)	34 (16)	29 (59)	12 (24)	10 (20)	75 (43)	30 (17)	25 (14)	21 (44)	15 (31)	4 (8)
Anemia^3^	63 (30)	13 (6)	0	26 (53)	7 (14)	0	40 (23)	8 (5)	0	14 (29)	3 (6)	0
Leukopenia^4^	33 (16)	19 (9)	2 (1)	8 (16)	4 (8)	1 (2)	18 (10)	8 (5)	2 (1)	7 (15)	2 (4)	0
Febrile neutropenia	11 (5)	10 (5)	1 (<1)	4 (8)	2 (4)	2 (4)	5 (3)	4 (2)	1 (1)	0	0	0
Gastrointestinal												
Diarrhea	121 (58)	22 (11)	0	32 (65)	5 (10)	0	20 (11)	1 (1)	0	7 (15)	0	0
Nausea	123 (59)	6 (3)	1 (<1)	24 (49)	0	0	48 (27)	0	0	11 (23)	1 (2)	0
Constipation	32 (15)	0	0	12 (24)	0	0	27 (15)	0	0	5 (10)	0	0
Vomiting	63 (30)	2 (1)	1 (<1)	12 (24)	0	0	20 (11)	0	0	3 (6)	1 (2)	0
Other												
Fatigue	91 (44)	6 (3)	0	24 (49)	2 (4)	0	49 (28)	10 (6)	0	19 (40)	2 (4)	0
Alopecia	101 (48)	0	0	18 (37)	0	0	27 (15)	0	0	8 (17)	0	0
Decreased appetite	40 (19)	2 (1)	0	11 (22)	2 (4)	0	25 (14)	1 (1)	0	7 (15)	0	0

*AE* adverse event, *MedDRA* Medical Dictionary for Regulatory Activities, *NCI CTCAE* National Cancer Institute Common Terminology Criteria for AE, *SG* sacituzumab govitecan, *TPC* treatment of physician’s choice, *TRAE* treatment-related AE.

^1^Patients may report more than 1 event per preferred term. AEs were coded using MedDRA v22.1, and AE severity was graded per NCI CTCAE v4.03.

^2^Combined preferred terms of “neutropenia” and “neutrophil count decreased.”

^3^Combined preferred terms of “anemia”, “hemoglobin decreased”, and “red blood cell count decreased.”

^4^Combined preferred terms of “leukopenia” and “white blood cell count decreased.”

### Efficacy by dose reductions and interruptions

Efficacy endpoints were assessed in the primary study analysis population of brain metastases-negative patients who received at least one dose of SG (10 mg/kg) or TPC. Dose interruptions occurred in 61% and 33% of patients in the SG and TPC arms, respectively, and dose reductions occurred in 22% and 26%, respectively. In the SG arm, efficacy outcomes for patients with dose reductions or interruptions were similar to those of patients without dose reductions or interruptions. SG treatment was associated with improved ORR, clinical benefit rate (CBR), and PFS compared with TPC in patients with and without dose interruptions, and with and without dose reductions (Table 3). Median PFS was 8.3 vs 2.9 months and 4.6 vs 1.5 months in SG- vs TPC-treated patients with and without dose reductions, respectively; it was 5.7 vs 2.7 months and 4.2 vs 1.6 months in SG- vs TPC-treated patients with and without dose interruptions, respectively.

**Table 3:** Efficacy outcomes for patients with dose reductions or interruptions (brain metastasis-negative population).

Outcome	Overall BMNeg population		Dose reductions		No dose reductions		Dose interruptions		No dose interruptions	
	SG (*n* = 235)	TPC (*n* = 233)	SG (*n* = 62)	TPC (*n* = 52)	SG (*n* = 173)	TPC (*n* = 181)	SG (*n* = 144)	TPC (*n* = 78)	SG (*n* = 91)	TPC (*n* = 155)
ORR (BICR), *n (%)*	82 (35)	11 (5)	29 (47)	7 (13)	53 (31)	4 (2)	56 (39)	5 (6)	26 (29)	6 (4)
CBR (BICR), *n (%)*	105 (45)	20 (9)	37 (60)	11 (21)	68 (39)	9 (5)	71 (49)	12 (15)	34 (37)	8 (5)
Best overall response, *n (%)*										
CR	10 (4)	2 (1)	5 (8)	1 (2)	5 (3)	1 (1)	7 (5)	0	3 (3)	2 (1)
PR	72 (31)	9 (4)	24 (39)	6 (12)	48 (28)	3 (2)	49 (34)	5 (6)	23 (25)	4 (3)
Median PFS (BICR), mo (95% CI)	5.6 (4.3–6.3)	1.7 (1.5–2.6)	8.3 (5.4–10.3)	2.9 (2.7–4.3)	4.6 (3.5–5.7)	1.5 (1.4–1.7)	5.7 (4.3–7.0)	2.7 (1.7–3.0)	4.2 (2.9–6.8)	1.6 (1.5–2.2)

Assessed in brain metastases-negative population (SG, *n* = 235; TPC, *n* = 233).

*BICR* blind independent central review, *BMNeg* brain metastases-negative, *CBR* clinical benefit rate, *CR* complete response, *ORR* objective response rate, *PFS* progression-free survival, *PR* partial response, *SG* sacituzumab govitecan, *TPC* treatment of physician’s choice.

### Safety by *UGT1A1* variant status

In the SG arm, *UGT1A1* variant status was known for 250 patients (97%), of whom 113 (44%), 96 (37%), and 34 (13%) had *1/*1 (wild type; normal activity), *1/*28 (heterozygous; reduced enzymatic activity), and *28/*28 (*28 homozygous; diminished enzymatic activity) genotypes, respectively (Table 1). The median SG relative dose intensity was 99.8%, 99.5%, and 99.8%, and mean time to first dose reduction was 2.7, 2.4, and 1.8 months in the wild type, heterozygous, and homozygous groups, respectively. TEAEs leading to dose reduction occurred in 18%, 19%, and 35% of patients in the wild type, heterozygous, and homozygous groups, respectively. Patients with the *28 homozygous genotype had a slightly higher rate of grade ≥3 treatment-related neutropenia (59%) than those with heterozygous (47%) and wild-type (53%) genotypes, but a considerably higher rate of treatment-related grade ≥3 febrile neutropenia (18% vs 5% and 3%, respectively) (Table 4). Grade ≥3 treatment-related anemia (15% vs 6% and 4%, respectively) and grade ≥3 treatment-related diarrhea (15% vs 9% and 10%, respectively) were also more common in patients with the *28 homozygous genotype. Other TRAEs, including nausea, vomiting, constipation, fatigue, alopecia, and decreased appetite, were not impacted by *UGT1A1* variant status. Treatment discontinuation due to TRAEs was more common in patients with the *28 homozygous genotype versus those with heterozygous and wild-type genotypes (6%, 1%, and 2%, respectively). The low frequency of the *28 homozygous polymorphism limited the ability to discern additional differences or draw any firm conclusions about differences in AEs.

**Table 4:** Key treatment-related adverse events of all grades in >20% and of grade ≥3 in >5% of patients treated with sacituzumab govitecan significantly impacted by UGT1A1 genotype.

TRAE^2^, *n* (%)	SG (*n* = 243)^1^					
	*1/*1 wild type (*n* = 113)		*1/*28 heterozygous (*n* = 96)		*28/*28 homozygous (*n* = 34)	
	All grades	Grade ≥ 3	All grades	Grade ≥ 3	All grades	Grade ≥ 3
Hematologic						
Neutropenia^3^	76 (67)	60 (53)	55 (57)	45 (47)	24 (71)	20 (59)
Anemia^4^	37 (33)	5 (4)	29 (30)	6 (6)	16 (47)	5 (15)
Leukopenia^5^	18 (16)	10 (9)	13 (14)	9 (9)	8 (24)	5 (15)
Lymphopenia^6^	10 (9)	1 (1)	5 (5)	1 (1)	4 (12)	2 (6)
Febrile Neutropenia	3 (3)	3 (3)	5 (5)	5 (5)	6 (18)	6 (18)
Thrombocytopenia^7^	3 (3)	0	6 (6)	0	4 (12)	4 (12)
Gastrointestinal						
Diarrhea	65 (58)	11 (10)	57 (59)	9 (9)	21 (62)	5 (15)

Assessed in the safety population of patients with UGT1A1 genotype. Shown are key TRAEs substantially impacted by the UGT1A1 *28/*28 genotype. Other TRAEs including nausea, vomiting, constipation, fatigue, alopecia, and decreased appetite were not substantially impacted.

*AE* adverse event, *NCI CTCAE* National Cancer Institute Common Terminology Criteria for AE, *SG* sacituzumab govitecan, *TRAE* treatment-related AE, *UGT* uridine diphosphate glucuronosyltransferase.

^1^Seven patients had UGT1A1 genotypes not listed in the table.

^2^Patients may report more than 1 event per preferred term. AEs were coded using MedDRA v22.1, and AE severity was graded per NCI CTCAE v4.03.

^3^Combined preferred terms of “neutropenia” and “neutrophil count decreased.”

^4^Combined preferred terms of “anemia” and “hemoglobin decreased.”

^5^Combined preferred terms of “leukopenia” and “white blood cell count decreased.”

^6^Combined preferred terms of “lymphopenia” and “lymphocyte count decreased.”

^7^Combined preferred terms of “thrombocytopenia” and “decreased platelet count.”

## Discussion

With results from the confirmatory phase 3 ASCENT study^25^, SG became the first ADC to demonstrate a significant survival improvement relative to standard single-agent chemotherapy in patients with pretreated mTNBC. Key AEs associated with SG included neutropenia and gastrointestinal toxicity, AEs expected with an SN-38 payload. The present analysis further elucidates the SG safety profile and AE management in patients with mTNBC, with special relevance to older individuals and those with *UGT1A1* *28 homozygous genotype, due to the potential role of *UGT1A1* genotype as a predictor of toxicity.

The safety profile of SG is consistent with previous reports^23,32,33^. In the overall study population (median age, 54 years; median of 4 prior anticancer regimens), TRAEs were primarily hematologic (neutropenia; grade ≥3, 51%) and gastrointestinal (diarrhea; grade ≥3, 10%). Use of supportive care medications was effective in managing these AEs, including myeloid growth factors and antipropulsive agents, and discontinuation due to toxicity was rare. Interestingly, in a comparison of this study population with the small subgroup of patients in the ASCENT trial who received SG in the second-line (2 L) metastatic setting (i.e., those who received only 1 line of therapy in the metastatic setting following recurrence ≤12 months after [neo]adjuvant chemotherapy; median of 3 prior anticancer regimens in any treatment setting, including the [neo]adjuvant setting), rates of key TRAEs were generally similar^34^. However, rates of SG-related all-grade alopecia were lower in the overall study population compared with that of the 2 L subgroup (Table 2; 46% vs 70%^34^). Because older age is a risk factor for chemotherapy toxicity, impacting treatment decisions and increasing the risk for AEs^35,36^, it is essential to understand the potential safety risks with SG in older patients. Irrespective of age, toxicities associated with SG were manageable, and the safety profile of SG in patients aged ≥65 and ≥75 years was generally similar to that of patients aged <65 years and the overall study population.

Of note, the observed delayed appearance of nausea and vomiting (median of 8 and 24.5 days after initiation of SG treatment, respectively) should be considered when managing treatment-associated symptoms. Patients should be provided with take-home medications for preventing and treating delayed nausea and vomiting, with clear instructions^26^. Antiemetics should be employed as clinically indicated, and atropine can be used to manage excessive cholinergic response^26^. Other AEs in the SG arm included alopecia (46%), which may affect quality of life^37^. Although therapeutic scalp cooling is effective for many chemotherapeutic agents, its efficacy with ADCs is unknown^38,39^. SG response and PFS advantage relative to TPC was maintained in patients who started at 10 mg/kg and then required SG dose reductions/interruptions to manage AEs; efficacy outcomes for patients with dose reduction or interruptions in the SG arm were similar to those for the overall study population^25^. In the ASCENT protocol, dose reduction/interruption recommendations for the TPC arm were based on local prescribing information or institutional standard practice, with no specific recommendations for myeloid growth factor use. A higher frequency of patients in the SG vs TPC arms received myeloid growth factors (49% vs 23%) and dose interruptions (61% vs 33%), which may have been due to heterogeneity in local guidelines for the different agents in the TPC arm; the frequency of patients with dose reductions (22% vs 26%, respectively) was similar. However, differences in the frequency of patients with dose reductions/interruptions should not affect the efficacy analysis for these groups, as there was a clinical benefit for SG in both patients with and without dose reductions/interruptions. These results support the recommended initial dosing of SG at 10 mg/kg followed by as-needed dose reductions and interruptions, over initiating treatment at a lower dose, which has the potential to compromise efficacy benefit^24^. Interestingly, median PFS was numerically higher in patients who received SG or TPC and had dose reductions/interruptions vs those who did not have dose reductions/interruptions. However, lack of pharmacokinetics, the small numbers of patients in these subgroup analyses, and the potential for confounding effects associated with dose reductions/interruptions limit further interpretation of these data.

Although hematologic toxicities are concerns for all the treatments administered in ASCENT, the safety profile of SG is distinct from those of eribulin, vinorelbine, capecitabine, and gemcitabine. Importantly, there were no instances of SG-related grade ≥3 neuropathy and low frequencies of SG-related interstitial lung disease (1 case of grade 3 pneumonitis, <1%), grade ≥3 rash (<1%), all-grade ocular disorders (<5%), and hyperglycemia (<2%); no SG-related deaths occurred. In comparison, peripheral neuropathy is a safety concern for eribulin, with 8% of patients who received single-agent eribulin in a metastatic breast cancer trial experiencing grade ≥3 events; peripheral neuropathy was also the most common adverse reaction resulting in discontinuation of eribulin (5%) in this trial^40^. Peripheral neuropathy is also a concern for single-agent vinorelbine, with clinical trial data showing 1% of patients experiencing grade ≥3 events^41^. Pulmonary toxicity, including fatal pneumonitis, has also been reported with vinorelbine^41^. Pulmonary toxicity and associated respiratory failure are also a major safety concern for gemcitabine^42^. In patients who receive single-agent capecitabine for metastatic breast cancer, a major safety concern is skin toxicity, with 57% and 11% experiencing capecitabine-related all-grade and grade 3 hand-and-foot syndrome, respectively, during clinical trials^43^.

Although not yet approved for TNBC, other ADCs being evaluated for breast cancer have distinct safety profiles. As with SG, common AEs with other ADCs include hematologic and gastrointestinal toxicities^44,45^. AEs associated with ADCs under investigation but not with SG include interstitial lung disease, neuropathy, ocular disorders, rash, and hyperglycemia^44,45^. These safety profile differences may be due in part to the payload of each ADC; with an SN-38 payload, SG has demonstrated a lower rate of diarrhea than that of SN-38’s prodrug, irinotecan^22^.

Our examination of potential *UGT1A1* polymorphism effects on SG toxicity was prompted by the enzyme’s known role in mediating the toxicity of SN-38, the cytotoxic species delivered to cells by SG. Clearance of SN-38, a lipophilic molecule, largely depends on its glucuronidation by *UGT1A1*, by which it is made water soluble for subsequent excretion^27^. In irinotecan-treated patients with colorectal cancer who have a *UGT1A1* polymorphism that limits SN-38 glucuronidation, increased SN-38 exposure results in higher rates of neutropenia and late-onset diarrhea^29,46^, the dose-limiting toxicities associated with irinotecan^47^. One such report found that patients receiving the combination of capecitabine and irinotecan with the *28 homozygous genotype were 14.2 times more likely to experience febrile neutropenia than those with the wild-type genotype^48^.

In line with other reports, the *28 homozygous genotype was found in a relatively small percentage of ASCENT patients (13%). As in previous irinotecan studies, rates of hematologic toxicities and diarrhea in ASCENT were modestly higher for patients with the *UGT1A1* *28 homozygous genotype than for those with the wild-type genotype. Specifically, rates of grade ≥3 neutropenia, febrile neutropenia, anemia, and diarrhea were higher in the *28 homozygous group compared with the heterozygous and wild-type groups. Despite this, the discontinuation rate for SG due to TRAEs for patients with the *28 homozygous genotype was low (6%), and no patients of any *UGT1A1* genotype discontinued SG due to SG-related neutropenia or diarrhea, suggesting favorable tolerability when using the current AE management strategies of active monitoring and early intervention (e.g., dose reductions and supportive medication use). These results imply that the currently recommended SG starting dosage (10 mg/kg) is appropriate regardless of patient *UGT1A1* genotype. Although this detailed safety analysis of ASCENT provides evidence of a manageable safety profile in patients with *UGT1A1* polymorphisms, the relatively low frequency of the *UGT1A1* *28 homozygous mutation limits the ability to discern differences or draw firm conclusions. Although prescreening for *UGT1A1* genotype is not required, individuals with known *UGT1A1* homozygous *28/*28 genotype should be monitored closely for neutropenia and diarrhea, regardless of age.

In summary, the results of this expanded ASCENT safety analysis show that SG has a manageable safety profile, irrespective of age, with an AE profile consistent with prior reports. These data confirm prior observations^24^, suggesting that *UGT1A1* variant status should not significantly alter recommendations for SG treatment or AE management in this patient population. Initial dosing at 10 mg/kg is recommended, with dose reductions as needed for toxicity, and these data suggest that dose reductions to manage toxicity do not appear to impact efficacy. Active monitoring, prophylaxis, and early intervention with routine AE management strategies like dose reductions allow for optimal therapeutic exposure of SG for patients with pretreated mTNBC.

## Methods

### Study design and participants

Detailed methods for ASCENT (NCT02574455; registered October 12, 2015), a phase 3 randomized, open-label multicenter study, have been reported^25^. ASCENT enrolled patients with mTNBC (per American Society of Clinical Oncology/College of American Pathologists criteria^49^ who relapsed after or were refractory to ≥2 previous standard chemotherapy regimens (no upper limit) for unresectable, locally advanced or metastatic disease. Patients whose disease recurred ≤12 months after completing (neo)adjuvant therapy were considered as having had one prior line of therapy. Screening for brain metastasis was not mandatory, and patients with brain metastases stable for ≥4 weeks before treatment were eligible. Patients were randomized 1:1 to SG 10 mg/kg intravenously on Days 1 and 8 of each 21-day cycle, or TPC (eribulin; vinorelbine; capecitabine; or gemcitabine) and received treatment until disease progression or unacceptable toxicity. Severe neutropenia and non-neutropenic toxicities were managed with dose delays or reductions and medications, as shown (Supplementary Fig. 2). Prophylactic growth factors at study start were not allowed, and antidiarrhea prophylaxis was not recommended.

Crossover to the SG arm was not allowed upon disease progression in the TPC arm. Patients were stratified by number of prior therapies (2–3 vs >3), geographic region (North America vs Europe), and the presence or absence of known brain metastasis (Yes or No). The primary endpoint was PFS in patients without known brain metastases by independent, centralized, and blinded review per Response Evaluation Criteria in Solid Tumors (RECIST), version 1.1. Secondary endpoints included safety. Exploratory endpoints included safety by *UGT1A1* variant status.

The study was approved by each institutional review board or ethics committee prior to initiation and was conducted in accordance with the Declaration of Helsinki and Good Clinical Practice Guidelines per the International Conference on Harmonization. A full list of institutions which granted ethical approval can be found in the Supplement. All patients provided written informed consent.

### Assessments

Response was evaluated as reported previously^50^. AEs were assessed at each study visit. Complete blood counts (CBC) and serum chemistries were performed at baseline and at last study visit for all treatments, and prior to infusion of SG or TPC agents administered intravenously (eribulin, gemcitabine, and vinorelbine), and at the start of each treatment cycle and as required per local standard of care for TPC agents administered orally (capecitabine). In the event of grade ≥3 hematologic toxicity, CBCs were obtained more frequently at physician’s discretion until toxicity recovered to baseline or grade 1. Descriptive statistics were used to characterize AEs. AEs were assessed per the National Cancer Institute Common Terminology Criteria for AEs, version 4.03, and coded per the Medical Dictionary for Regulatory Activities, version 22.1. At baseline, a single whole blood sample was collected from all patients receiving SG for determination of *UGT1A1* genotype for retrospective assessment of safety. *UGT1A1* genotype was determined by Sanger sequencing, performed centrally. The relationship between the incidence of AEs of special interest to SG, including hematologic events and diarrhea, and *UGT1A1* genotype were of particular focus.

### Statistical analysis

The safety population consisted of all patients who received ≥1 dose of study drug. Relative dose intensity was calculated by dividing the cumulative dosage received (in mg/kg) by the product of the assigned dose (in mg/kg) based on the number of doses the patient was scheduled to receive during the patient’s treatment period. Time to onset of first AEs was defined as time from first dosing date to the start date of the AE. Duration of an individual episode of AE was calculated as the AE end-date minus the AE start-date. Median of the individual episodes of AE was used for calculation at the population level for patients with multiple episodes. PFS was defined as time from randomization until objective tumor progression or death or censored at last radiographic assessment for patients without progression or death. PFS was analyzed by the Kaplan-Meier method; 95% CIs were determined according to the method of Brookmeyer and Crowley.

### Supplementary information

Supplementary Material

## Data Availability

Gilead Sciences shares anonymized individual patient data upon request or as required by law or regulation with qualified external researchers based on submitted curriculum vitae and reflecting non conflict of interest. The request proposal must also include a statistician. Approval of such requests is at Gilead Science’s discretion and is dependent on the nature of the request, the merit of the research proposed, the availability of the data, and the intended use of the data. Data requests should be sent to datarequest@gilead.com.
