# Author Correction: Safety analyses from the phase 3 ASCENT trial of sacituzumab govitecan in metastatic triple-negative breast cancer

**DOI:** 10.1038/s41523-024-00650-6

**Published:** 2024-06-06

**Authors:** Hope S. Rugo, Sara M. Tolaney, Delphine Loirat, Kevin Punie, Aditya Bardia, Sara A. Hurvitz, Joyce O’Shaughnessy, Javier Cortés, Véronique Diéras, Lisa A. Carey, Luca Gianni, Martine J. Piccart, Sibylle Loibl, David M. Goldenberg, Quan Hong, Martin Olivo, Loretta M. Itri, Kevin Kalinsky

**Affiliations:** 1 Department of Medicine, University of California San Francisco Helen Diller Family Comprehensive Cancer Center, San Francisco, CA USA; 2 Department of Medical Oncology, Dana-Farber Cancer Institute, Boston, MA USA; 3 Department of Medical Oncology and D3i, Institut Curie, Paris, France; 4 Department of General Medical Oncology, University Hospitals Leuven, Leuven, Belgium; 5 Department of Hematology/Oncology, Massachusetts General Hospital Cancer Center, Harvard Medical School, Boston, MA USA; 6 Medical Oncology, University of California, Los Angeles, Jonsson Comprehensive Cancer Center, Los Angeles, CA USA; 7 Baylor University Medical Center, Texas Oncology, US Oncology, Dallas, TX USA; 8 International Breast Cancer Center (IBCC), Quiron Group, Madrid & Barcelona, Barcelona, Spain; 9 Vall d´Hebron Institute of Oncology (VHIO), Barcelona, Spain; 10 Universidad Europea de Madrid, Faculty of Biomedical and Health Sciences, Department of Medicine, Madrid, Spain; 11 Department of Medical Oncology, Centre Eugène Marquis, Rennes, France; 12 Department of Hematology and Oncology, University of North Carolina Lineberger Comprehensive Cancer Center, Chapel Hill, NC USA; 13 Gianni Bonadonna Foundation, Milano, Italy; 14 Medical Oncology, Institute Jules Bordet, Brussels, Belgium; 15 Department of Medicine and Research, Hämatologisch-Onkologische Gemeinschaftspraxis am Bethanien-Krankenhaus, Frankfurt, Germany; 16 Department of Clinical Development, Immunomedics, Inc., Morris Plains, NJ USA; 17 Columbia University Irving Medical Center, New York, NY USA; 18 Present Address: Winship Cancer Institute, Emory University, Atlanta, GA USA

**Keywords:** Breast cancer, Drug safety

Correction to: *npj Breast Cancer* (2022) **8**:98; 10.1038/s41523-022-00467-1, published online 29 August 2022

The article includes two typographical errors, on pages 5 and 6. On page 5, in the sentence beginning with “Dose interruptions…”, and on page 6, in the sentence beginning with “A higher frequency…”, the order of the percentage of patients in the SG and TPC arms who discontinued treatment due to adverse events in the ASCENT trial has been written backwards. Therefore, on page 5, values “26% and 22%, respectively” have been corrected to “22% and 26%, respectively”. Similarly, on page 6, values “(26% vs. 22%, respectively)” have been corrected to “(22% vs. 26%, respectively)”.

