# Supplementary material for: Safety analyses from the phase 3 ASCENT trial of sacituzumab govitecan in metastatic triple-negative breast cancer
Source: NPJ Breast Cancer. 2022 Aug 29;8:98. doi: 10.1038/s41523-022-00467-1 (PMC9424318; doi:10.1038/s41523-022-00467-1)
Supplement: Supplementary file 1 — Supplementary Material [file 41523_2022_467_MOESM1_ESM.pdf]

Safety analyses from the phase 3 ASCENT trial of sacituzumab govitecan in metastatic triple-negative breast cancer

Rugo et al.

SUPPLEMENTARY APPENDIX

Table of Contents **Page Numbers**

Table of Contents	Page Numbers
**List of institutions which provided ethical approval for ASCENT**	**2**
**Supplementary Table 1.** Treatment-related adverse events (all grade, >20%; grade 3 or 4, >5% of patients) by sacituzumab govitecan and treatment of physician's choice agents	**4**
**Supplementary Table 2.** Summary of adverse events by age	**6**
**Supplementary Figure 1.** ASCENT CONSORT diagram: Enrollment, intent-to-treat and safety populations, and patient withdrawals	**8**
**Supplementary Figure 2.** Adverse event management strategy for sacituzumab govitecan	**10**
**Supplementary References**	**12**

List of institutions which provided ethical approval for ASCENT:

Allegheny-Singer Research Institute, Pittsburgh, PA, USA; Barts Cancer Institute, Queen Mary University of London, London, United Kingdom; Beth Israel Deaconess Medical Center , Boston, MA, USA; Blue Ridge Cancer Care, Salem, VA, USA; Centre Eugène Marquis, Rennes, France; Centre Léon Bérard, Lyon, France; CHU Besançon, Hôpital Jean Minjoz, Besançon, France; CHU UCL Namur, Site Sainte Elisabeth, Namur, Belgium; Columbia University Irving Medical Center, New York, NY, USA; Complexo Hospitalario Universitario de Santiago (CHUS) – Hospital Clínico Universitario, A Coruña, Spain; Cross Cancer Institute, Edmonton, Alberta, Canada; Dana-Farber Cancer Institute, Boston, MA, USA; Facharztzentrum Eppendorf, Hamburg, Germany; Florida Cancer Specialists & Research Institute, Daytona Beach, FL, USA; Florida Cancer Specialists South, Fort Myers, FL, USA; Florida Cancer Specialists, Tampa, FL, USA; Georgetown Lombardi Comprehensive Cancer Center, Washington, DC, USA; Gianni Bonadonna Foundation, Milano, Italy; Hämatologisch-Onkologische Gemeinschaftspraxis am Bethanien-Krankenhaus, Frankfurt, Germany; Hospital del Mar, Barcelona, Spain; Hospital Teresa Herrera, A Coruña, Spain; Hospital Universitario 12 de Octubre, Madrid, Spain; Hospital Universitario Ramón y Cajal, Madrid, Spain; Hospital Universitario Virgen del Rocío, Sevilla, Spain; Illinois Cancer Specialists, Niles, IL, USA; Institut Catala d'Oncologia Hospitalet, Barcelona, Spain; Institut Claudius Regaud, IUCT – Oncopole, Toulouse, France; Institut Curie – Saint-Cloud, Saint-Cloud, France; Institut Curie, Paris, France; Institut de Cancérologie de l'Ouest (Site René Gauducheau), Saint-Herblain, France; Institut de Cancérologie de l'Ouest (Site René Gauducheau), Villejuif, France; Institut Gustave Roussy, Villejuif, France; Institut Jules Bordet, Université libre de Bruxelles, Brussels, Belgium; Institut Régional du Cancer de Montpellier, Montpellier, France; Instituto Oncológico Baselga – Hospital Quirónsalud Barcelona, Barcelona, Spain; International Breast Cancer Center (IBCC), Quiron Group, Barcelona, Spain; Leuven Cancer Institute, University Hospitals Leuven, Leuven, Belgium; Magee-Women's Hospital and the Hillman Cancer Center, University of Pittsburgh Medical Center, Pittsburgh, PA, USA; Maryland Oncology Hematology – Clinton Office, Clinton, MD, USA; Massachusetts General Hospital Cancer Center, Harvard Medical School, Boston, MA, USA; Mayo Clinic, Rochester, MN, USA; Memorial Sloan Kettering Cancer Center, New York, NY, USA; Methodist Hospital, Houston, TX, USA; Miami Cancer Institute and Baptist Health South Florida, Miami, FL, USA; New York Oncology Hematology, PC, Albany, NY, USA; North Shore Hematology Oncology Associates, PC, Patchogue, NY, USA; Northside Hospital, Atlanta, GA, USA; Norwalk Hospital, Norwalk, CT, USA; Orlando Regional Medical Center, Orlando, FL, USA; Praxisklinik für Hämatologie und Onkologie Koblenz, Koblenz, Germany; Providence Cancer Center, Portland, OR, USA; Queen Elizabeth II Health Sciences Centre, Halifax, Nova Scotia, Canada; Rocky Mountain Cancer Centers, Greenwood Village, CO, USA; Rutgers Cancer Institute of New Jersey, New Brunswick, NJ, USA; Sarah Cannon Research Institute – Research Medical Center Kansas City, Kansas City, MO, USA; Segal Cancer Centre, Jewish General Hospital, Montreal, Quebec, Canada; Southern Cancer Center, Daphne, AL, USA; Surrey and Sussex Healthcare NHS Trust, East Surrey Hospital, Surrey, United Kingdom; Swedish Cancer Institute, Chicago, IL, USA; Sylvester Comprehensive Cancer Center, Plantation, FL, USA; Taunton and Somerset NHS Foundation Trust – Musgrove Park Hospital, Taunton, United Kingdom; Tennessee Oncology – Chattanooga, Chattanooga, TN, USA; Tennessee Oncology, Nashville, TN, USA; Texas Oncology – Baylor Charles A. Sammons Cancer Center, Dallas, TX, USA; Texas Oncology – Denton, Denton, TX, USA; Texas Oncology – Plano East, Plano, TX, USA; The Center for Cancer and Blood Disorders, Fort Worth, TX, USA; The Ohio State University Wexner Medical Center, Columbus, OH, USA; The Royal Free London NHS Foundation Trust – The Royal Free Hospital, London,

United Kingdom; The University of Chicago Medical Center, Chicago, IL, USA; Universitair Ziekenhuis Brussel, Brussels, Belgium; University Cancer & Blood Center, Athens, GA, USA; University Hospitals Coventry and Warwickshire NHS Trust, Coventry, United Kingdom; University of California San Francisco Helen Diller Family Comprehensive Cancer Center, San Francisco, CA, USA; University of California, Los Angeles, Jonsson Comprehensive Cancer Center, Los Angeles, CA, USA; University of Colorado Hospital – Anschutz Cancer Pavilion, Aurora, CO, USA; University of Kansas Cancer Center, Westwood, KS, USA; University of North Carolina Lineberger Comprehensive Cancer Center, Chapel Hill, NC, USA; US Oncology Research Pharmacy, Texas Oncology – Tyler, Tyler, TX, USA; Vall d'Hebron University Hospital and Vall d'Hebron Institute of Oncology (VHIO), Barcelona, Spain; Vanderbilt-Ingram Cancer Center, Nashville, TN, USA; Virginia Cancer Specialists, PC, Fairfax, VA, USA; Virginia Oncology Associates, PC, Norfolk, VA, USA; Virginia Piper Cancer Institute Oncology Research, Minneapolis, MN, USA; Washington University School of Medicine in St. Louis, St Louis, MO, USA; West Cancer Center, Memphis, TN, USA.

Supplementary Table 1 Treatment-related adverse events (all grade, >20%; grade 3 or 4, >5% of patients) by sacituzumab govitecan and treatment of physician's choice agents

TRAE[a], *n (%)*	SG (*n*=258) [1]		TPC (*n*=224) Eri (*n*=123)		TPC (*n*=224) Vin+Cape+Gem (*n*=101)		TPC (*n*=224) Eri+Vin+Cape+Gem (*n*=224)[1]	
	**All grades**	**Grade 3/4**	**All grades**	**Grade 3/4**	**All grades**	**Grade 3/4**	**All grades**	**Grade 3/4**
Hematologic								
Neutropenia[b]	163 (63)	132 (51)	48 (39)	38 (31)	48 (48)	36 (36)	96 (43)	74 (33)
Anemia[c]	89 (34)	20 (8)	28 (23)	3 (2)	26 (26)	8 (8)	54 (24)	11 (5)
Leukopenia[d]	41 (16)	26 (10)	14 (11)	6 (5)	11 (11)	6 (6)	25 (11)	12 (5)
Febrile neutropenia	15 (6)	15 (6)	3 (2)	3 (2)	2 (2)	2 (2)	5 (2)	5 (2)
Gastrointestinal								
Diarrhea	153 (59)	27 (10)	10 (8)	0	17 (17)	1 (1)	27 (12)	1 (<1)
Nausea	147 (57)	7 (3)	36 (29)	1 (1)	23 (23)	0	59 (26)	1 (<1)
Vomiting	75 (29)	3 (1)	14 (11)	1 (1)	9 (9)	0	23 (10)	1 (<1)
Other								
Alopecia	119 (46)	0	31 (25)	0	4 (4)	0	35 (16)	0
Fatigue	115 (45)	8 (3)	38 (31)	6 (5)	30 (30)	6 (6)	68 (30)	12 (5)

[a]Patients may report more than 1 event per preferred term. AEs were coded using MedDRA v22.1, and AE severity was graded per NCI CTCAE v4.03.

[b]Combined preferred terms of "neutropenia" and "neutrophil count decreased." Due to overlapping reporting of events for these combined terms, all grades reported are not shown for the SG arm: Grade 1: 19%; Grade 2: 37%. [c]Combined preferred terms of "anemia", "hemoglobin decreased", and "red blood cell count decreased." [d]Combined preferred terms of "leukopenia" and "white blood cell count decreased."

Abbreviations: AE, adverse event; Cap, capecitabine; Eri, eribulin; Gem, gemcitabine; MedDRA, Medical Dictionary for Regulatory Activities; NCI CTCAE, National Cancer Institute Common Terminology Criteria for AE; SG, sacituzumab govitecan; TPC, treatment of physician's choice; TRAE, treatment-related AE; Vin, vinorelbine.

Supplementary Table 2 Summary of adverse events by age

	SG			TPC		
**Event, *n (%)***	**<65 years** (*n*=209)	**≥65 years** (*n*=49)	**≥75 years** (*n*=8)	**<65 years** (*n*=176)	**≥65 years** (*n*=48)	**≥75 years** (*n*=13)
**Any TEAE[a]**	208 (99.5)	49 (100)	8 (100)	171 (97)	48 (100)	13 (100)
Grade ≥3	153 (73)	33 (67)	6 (75)	115 (65)	30 (63)	8 (62)
Leading to dose reduction	39 (19)	17 (35)	1 (13)	43 (24)	16 (33)	4 (31)
Leading to study drug discontinuation	11 (5)	1 (2)	0	11 (6)	1 (2)	1 (8)
**Any TRAE**	204 (98)	48 (98)	8 (100)	152 (86)	40 (83)	12 (92)
Grade ≥3	135 (65)	31 (63)	6 (75)	79 (45)	26 (54)	7 (54)
Leading to dose reduction	39 (19)	17 (35)	1 (13)	41 (23)	16 (33)	4 (31)
Leading to study drug discontinuation	4 (2)	1 (2)	0	6 (3)	0	0
Leading to death	0	0	0	1 (1)	0	0

TEAE is defined as an adverse event with start date on or after the date of first dose of study treatment and up to 30 days after date of last dose of study treatment.

[a]Patients may report more than 1 event per preferred term. AEs were coded using MedDRA v22.1, and AE severity was graded per NCI CTCAE v4.03.

Abbreviations: AE, adverse event; MedDRA, Medical Dictionary for Regulatory Activities; NCI CTCAE, National Cancer Institute Common Terminology Criteria for AE; SG, sacituzumab govitecan; TPC, treatment of physician's choice; TEAE, treatment-emergent adverse event; TRAE, treatment-related AE

Supplementary Figure 1

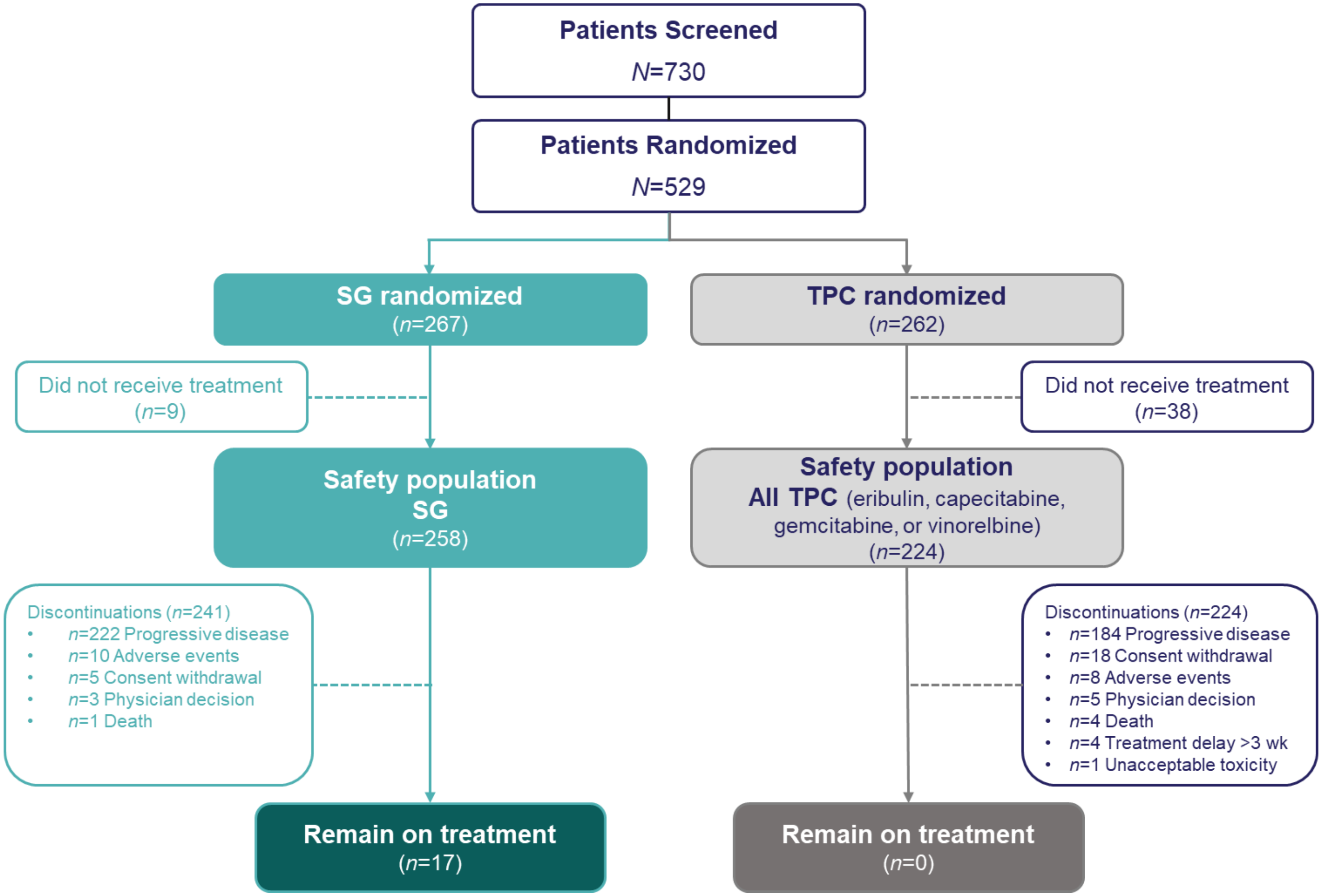

Supplementary Figure 1: ASCENT CONSORT diagram: Enrollment, intent-to-treat and safety populations, and patient withdrawals.

Patients in the safety population received at least 1 dose of SG or TPC. Patients in the TPC arm received eribulin (*n*=123), vinorelbine (*n*=41), gemcitabine (*n*=32), or capecitabine (*n*=28).

Abbreviations: SG, sacituzumab govitecan; TPC, treatment of physician's choice.

Supplementary Figure 2

Severe Neutropenia

- Grade 4 neutropenia ≥7 days, ***OR***
- Grade 3 febrile neutropenia (absolute neutrophil count <1000/mm^3 and fever ≥38.5°C), ***OR***
- At time of scheduled treatment, grade 3-4 neutropenia which delays dosing by 2 or 3 weeks for recovery to ≤ grade 1

1st occurrence → 25% dose reduction and administer G-CSF

2nd occurrence → 50% dose reduction

3rd occurrence → Discontinue treatment

- At time of scheduled treatment, grade 3-4 neutropenia, which delays dosing beyond 3 weeks for recovery to ≤ grade 1

1st occurrence → Discontinue treatment

Severe Non-Neutropenic Toxicity

- Grade 4 non-hematologic toxicity of any duration, ***OR***
- Any grade 3-4 nausea, vomiting or diarrhea due to treatment that is not controlled with antiemetics and anti-diarrheal agents, ***OR***
- Other grade 3-4 non-hematologic toxicity persisting >48 hours despite optimal medical management, ***OR***
- At time of scheduled treatment, grade 3-4 non-neutropenic hematologic or non-hematologic toxicity, which delays dose by 2 or 3 weeks for recovery to ≤ grade 1

1st occurrence → 25% dose reduction

2nd occurrence → 50% dose reduction

3rd occurrence → Discontinue treatment

- In the event of grade 3-4 non-neutropenic hematologic or non-hematologic toxicity, which does not recover to ≤ grade 1 within 3 weeks

1st occurrence → Discontinue treatment

- Onset of severe diarrhea

Management →

- Evaluate for infectious cause
- If negative, promptly initiate loperamide 4 mg then 2 mg with every diarrhea episode, max of 16 mg/d daily
- Discontinue loperamide 12 hours after diarrhea resolves

Supplementary Figure 2 Adverse event management strategy for sacituzumab govitecan.

The recommendation for 25% dose reduction alongside administration of G-CSF upon first occurrence of severe neutropenia presented in this figure are based on United States regulatory agency modifications to the AE management guidelines for sacituzumab govitecan in the US PI[2] and differ from those in the ASCENT study protocol (administration of G-CSF recommended, without dose reduction). European Union guidelines for the first occurrence of severe neutropenia correspond to those in the ASCENT study protocol and only recommend administration of G-CSF, without dose reduction.[3]

From *The New England Journal of Medicine*, Bardia A, Hurvitz SA, Tolaney SM, et al. Sacituzumab govitecan in metastatic triple-negative breast cancer. Vol. 384, pp 1529-1541. Copyright ©2021 Massachusetts Medical Society. Reprinted with permission from Massachusetts Medical Society.

Abbreviation: AE, adverse event; G-CSF, granulocyte colony-stimulating factor; US PI, United States prescribing information.

SUPPLEMENTARY REFERENCES

1 Bardia, A. *et al.* Sacituzumab govitecan in metastatic triple-negative breast cancer. *N Engl J Med* **384**, 1529-1541, doi:10.1056/NEJMoa2028485 (2021).

2 TRODELVY (sacituzumab govitecan-hziy). Prescribing information. Gilead Sciences, Inc.; 2021.

3 TRODELVY (sacituzumab govitecan). Summary of product characteristics. Gilead Sciences; 2021.
